# The Influence of Intersections on Fuel Consumption in Urban Arterial Road Traffic: A Single Vehicle Test in Harbin, China

**DOI:** 10.1371/journal.pone.0137477

**Published:** 2015-09-14

**Authors:** Lina Wu, Yusheng Ci, Jiangwei Chu, Hongsheng Zhang

**Affiliations:** 1 College of Automobile and Traffic Engineering, Heilongjiang Institute of Technology, Harbin, Heilongjiang, China; 2 College of Traffic, Northeast Forestry University, Harbin, Heilongjiang, China; 3 School of Transportation Science and Engineering, Harbin Institute of Technology, Harbin, Heilongjiang, China; 4 School of Mechatronics Engineering, Harbin Institute of Technology, Harbin, Heilongjiang, China; University of California Berkeley, UNITED STATES

## Abstract

The calculating method for fuel consumption (*FC*) was put forward and calibrated and the characteristics of the fuel consumption on intersections were analyzed based on 18 groups of vehicular operating data which were acquired from the test experiment of a single vehicle along the urban arterial roads in Harbin, China. The results obtained show that 50.36% of the fuel consumption for the test vehicle was used at the area of intersections compared with 28.9% of the influencing distance and 68.5% of the running time; and 78.4% of them was burnt at the stages of acceleration and idling. Meanwhile, the type (c) of the vehicular operating status was illustrated to be the worst way of reducing fuel consumption, the causes were analyzed and four improvement strategies were put forward.

## Introduction

Statistically, one-third of the gasoline was exhausted by autos worldwide each year; and energy supply had become one of huge challenges for human beings. Consequently, 3 main fuel consumption reduction technologies were put forward and implemented from the aspects of vehicles, driving behaviors and traffic management and control respectively. Above all, advanced engine technologies, which were usually improved by auto makers and researchers, were gradually applied including hybrid electric engine, and turbocharger technology. For instance, hybrid electric vehicles (HEVs) or plug-in hybrid electric vehicles (PHEVs) were proved to be successful to reduce the fuel consumption; many relative strategies and methods were proposed to save the HEVs fuel consumption. Hu *et al*. and Yang *et al*. addressed the electrochemical energy buffers and robust coordinated control applied for the HEVs separately and the results showed that the proposed strategy could improve the fuel economy [1,2]. Hu *et al*. and Li *et al*. discussed the two optimization-based and dynamic programming-based global optimal energy management strategies for the plug-in hybrid electric bus (PHEBs) [3,4]; Sun *et al*. developed a PHEVs energy management algorithm to achieve enhanced fuel economy under different traffic flow information conditions [5]. Zheng *et al*. analyzed the effect of battery temperature on fuel consumption of fuel cell hybrid vehicles (FCHVs) [6]; Hu *et al*. discussed the optimal dimensioning or longevity-conscious dimensioning and power management for the fuel cell hybrid buses (FCHBs) with the method of convex programming and the findings showed that they are optimal and efficient [7,8]. Meanwhile, stop-start technology (STT) can save about 5~15% fuel consumption [9]; and the turbocharger technology can also reduce fuel consumption for the vehicles and be used widely.

Then, eco-driving is encouraged and can reduce fuel consumption accordingly. Of course, some assistance system should be used. Staubach *et al*. evaluated an eco-driving support system utilizing a driving simulator; the system can communicate with traffic lights and gives recommendations to the drivers concerning acceleration/deceleration behaviors [10]. Lai discussed the effects of eco-driving on fuel efficiency with the before-after data with a reward system; and the results indicated that around 10% of the fuel consumption can be saved with the system [11]. McIlroy *et al*. provided a decision ladder analysis of eco-driving and a discussion of the resultant models for the skills, rules and knowledge taxonomy [12].

Finally, the strategies and methods of traffic management and control can be implemented to economize fuel consumption. This is the focus of this article. A number of earlier research findings revealed that the fuel consumption per unit distance can be approximately described as a linear function of the average speed and stop frequency [13,14]. Rakha *et al*. indicated the vehicle fuel consumption rates increased considerably as a stop, especially at high cruising speeds. Meanwhile, the levels of average speed and acceleration were also of significant importance [15]. Ahn *et al*. built up microscopic fuel consumption and emission models with the input variables of instantaneous vehicle speed and acceleration levels and developed using the Oak Ridge National Laboratory (ORNL) data [16].

However, the fuel consumption on intersection area is completely different with common urban road segments. Here are 3 primary kinds of advanced control strategies we can conclude. For a start, traffic engineers or researchers put forward relative methods to analyze or reduce the fuel consumption on intersection involving vehicle running state analysis, traffic design, traffic management and control and traffic flow investigation. Luo set up vehicle fuel consumption models based on vehicle running state data by simulating at intersections. It shows that the fuel consumption is mainly affected by travel speed, stop rate and delay [17]. Zhang *et al*. and Xiang *et al*. discussed the factors affecting vehicle fuel consumption, analyzed the vehicle running characteristics and proposed fuel consumption models at pre-time signalized intersections [18,19]. Feng *et al*. established a statistical model for evaluation on fuel economy of urban intersections considering saturation and splits and revealed the relationship among the traffic flow state, signal control parameters and the fuel economy [20]. Moreover, connected vehicles are being used to reduce fuel consumption, such as vehicle to infrastructure (V2I), vehicle to vehicle (V2V) and etc. Adaptive traffic lights using car-to-car communication is a manner of great of importance. Li *et al*. put forward the concept of safety driving patterns, analyzed the group communication strategy at the blind crossings and finally conducted simulation studies [21]. Asadi *et al*. and Mahler *et al*. presented the use of upcoming traffic signal information and a predictive optimal velocity-planning algorithm to reduce idle time and fuel consumption for the vehicles at the stop lines [22,23]; Bie *et al*. proposed a travel time prediction model on be basis of global positioning system (GPS) data [24]. Finally, eco-driving has gained interest worldwide. Xia *et al*. developed a dynamic eco-driving velocity planning method and the corresponding algorithm for the signalized arterial corridors and it approved that it can save around 10–15% of the fuel consumption [25]; and Zhang *et al*. proposed the eco-driving strategies and the model with good performance on energy consumption reduction [26].

Unfortunately, little information has focus on the vehicle fuel consumption influenced by the existence of the intersection in urban arterial road directly. It has been approved that the impact of the intersection on the vehicular operating is significant in the urban arterial road. How does it affect the fuel consumption of the vehicle exactly? This paper tries to use the detailed on-site test to illustrate this point. Therefore, the core interest of this paper lies in problems concerning the composing, calculating method and characteristics of the fuel consumption for the intersections of urban arterial roads in Harbin, China.

The paper consists of following sections: Section 2 shows the data acquisition. Section 3 builds fuel consumption calculation model and its calibration; Section 4 analyses the characteristics of fuel consumption on intersections; Section 5 is the discussion; and the conclusions are drawn in Section 6.

## Data Acquisition

### Test route and test vehicle

#### Test route

Origin: 2 Huashan North Road, Nangang District, Harbin, China.

Destination: 228 Dongzhi Road, Daowai District, Harbin, China.

Road composing: 4 arterial roads including Huashan North Road, Xianfeng Road, Hongqi Avenue and Dongzhi Road.

Intersection types and composition: 10 intersections including 9 signalized intersections and a 3-way stop intersection.

Route distance: the whole route is 4370 meters long.

Pavement condition: the roadway pavements of the entire route are paved with asphalt concrete within 5 years and dry, and there are no significant ruts and other distresses.

The test route is designed and showed (S1 Fig).

#### Test vehicle

A gasoline car of 2003 Jetta CIF with 138,000 km and a 2011 Skoda Octavia with 72,490 km were used as the test vehicles and were both timely maintained. Meanwhile, there was a driver and a passenger in the car without any other loading. The relative technical parameters of the test vehicles are listed (S1 Table).

### *FC* test system

A software named VCDS ZHS 12.12.0 was designed by Ross-Tech LLC, which is a diagnostic and test system for VW-Audi group cars. Here, it was installed at the computer and connected with the test car using the given cable correctly before testing. The operation interface and the cable of the test system are showed (S2 Fig).

From the test system, the data of engine velocity, engine load, injection timing, recording time and etc. were recorded as a Microsoft excel file.

### Sampling

Along the test route, the experiments were implemented during the non-peak hours, morning and evening rush hours on October 11^th^, 13^th^, 14^th^, 16^th^, 21^st^, 22^nd^, and 27^th^, 2014 and July 1^st^, and 2^nd^, 2015. Consequently, 18 groups of experimental data were acquired by the test system.

## *FC* Calculation

### *FC* composing

*FC* of the test vehicle is composed of the following four parts, which are the acceleration (*FC*_*a*_), the deceleration *FC* (*FC*_*d*_), the idling *FC* (*FC*_*i*_) and the uniform velocity travelling *FC* (*FC*_*u*_).

$$FC=FC_{a}+FC_{d}+FC_{i}+FC_{u}$$ (1)

$$FC_{a}=\sum_{i=1}^{n_{a}}\int_{0}^{t}t_{a}f_{a}dt=\sum_{i=1}^{n_{a}}f_{a}\int_{0}^{t}t_{a}dt=n_{a}t_{0}f_{a}$$ (2)

$$FC_{d}=\sum_{i=1}^{n_{d}}\int_{0}^{t}t_{d}f_{d}dt=\sum_{i=1}^{n_{d}}f_{d}\int_{0}^{t}t_{d}dt=n_{d}t_{0}f_{d}$$ (3)

$$FC_{i}=\sum_{i=1}^{n_{i}}\int_{0}^{t}t_{i}f_{i}dt=\sum_{i=1}^{n_{i}}f_{i}\int_{0}^{t}t_{i}dt=n_{i}t_{0}f_{i}$$ (4)

$$FC_{u}=\sum_{i=1}^{n_{u}}\int_{0}^{t}t_{u}f_{u}dt=\sum_{i=1}^{n_{u}}f_{u}\int_{0}^{t}t_{u}dt=n_{u}t_{0}f_{u}$$ (5)

Where, *t*_*a*_, *t*_*d*_, *t*_*i*_ and *t*_*u*_ are the average acceleration time, the average deceleration time, the average idling time and the uniform velocity travelling time respectively (s); *t* is the time variable (s); *t*_0_ is the record time interval (s); *f*_*a*_, *f*_*d*_, *f*_*i*_ and *f*_*u*_ are the corresponding average *FC* rates (*FCR*s) severally (ml/s); and *n*_*a*_, *n*_*d*_, *n*_*i*_ and *n*_*u*_ are the recorded segment counts.

### *FCR*s calibration

From above analysis, *FCR*s are the decisive factors to computing *FC*. *FCR*s calibration hereby was done as follows.

First, the fuel injection quantity for the *i* ^th^ time interval is computed as eq (6).

$$q_{i}=\frac{t_{0}}{t_{e}}\times (\frac{\tau_{i-1}+\tau_{i}}{2})\times C\times K\times \frac{1}{d}$$ (6)

Where, *t*_*e*_ is calculated as eq (7).

$$t_{e}=\frac{2\times 60}{n_{e}}$$ (7)

Where, *q*_*i*_ is the fuel injection quantity for the *i* ^th^ time interval (ml); *τ*_*i*_ is the injection pulse width for the *i* ^th^ record (ms); *C* is the number of cylinders; *K* is the calibrated parameter of the fuel injection quantity, 0.003 g/s; *t*_*e*_ is the injection time interval (s); *n*_*e*_ is the engine velocity (r/min); and *d* is the density of the used gasoline (g/ml).

Second, *FCR*s are computed as follows.

$$f_{j}=\sum_{i=1}^{n_{j}}q_{i}/t_{0}$$ (8)

Where, the values of *j* are *a*, *d*, *i*, and *u*.

According to the collected data, average *FCR*s were calibrated (S2 Table).

The *FCR*s calibration results for the test vehicle shows that: *f*_a_ is the highest and it’s twice more of *f*_u_ and four times more of *f*_d_ and *f*_i_; *f*_i_ is the lowest and with little gap of *f*_d_.

### *FC* test results

According to the above equations, the real *FC*s for the test vehicle along the test route was calculated based on the collected data (S3 Table).

The average *FC* of the test vehicle for the test route is 521.11 ml and the conversion of *FC* per hundred kilometers is 11.9 l/100 km (S3 Table).

## Characteristics of *FC*s on Intersections

### Intersection influencing distance

The intersection influencing distance (IID) is defined as the area range that the vehicle operation is influenced by the existence of the intersection, which consists of two components, the former is the distance between the stop lines of the approach and exit of the intersection along the test route direction, it’s a constant; and the latter is the dilemma zone or the distance between the stop line of the approach and the location of the first stop in the intersection area, it’s a variable.

From the on-site measurement, the first part of the IIDs is 394 meters. However, the second part of the IIDs is varied from 658 meters to 1,200 meters and the average distance is 868 meters. Accordingly, the average is 1,262 meters occupying 28.9% of the whole distance of the test route.

### Intersection operation time

The intersection operation time (IOT) is defined as the time duration of the test vehicle travelling during IID. The intersection operation time varies from 392 seconds to 1051 seconds and the average value is 576 seconds accounting for 68.5% of the total test time.

Here, a space-time diagram of the test vehicle from the collected data is showed (S3 Fig).

In this space-time diagram of the test route, the main part of test time is made up of IOTs. Furthermore, key intersections can be identified as influencing the operation time and *FC*.

### Characteristics of *FC*s on intersections

From the data collected by the test system, *FC*s on intersections can be computed (S4 Table).

From the above statistical data, several findings can be discovered as the result of analysis.

For one thing, *FC*s on intersections vary from 190.41 ml to 370.25 ml and the average value is 262.43 ml; it will be much clearer in this way, the percentages of the *FC*s on intersections in *FC*s are from 42.35% to 59.93% and the average percentage is 50.36%, and it means that more than half of the *FC* was used at the area of intersections compared with 28.9% of the IIDs.

For the other, the average *FC*_a_ is 109.05 ml accounting for 41.6% of the average *FC* on intersections and which is the most important part; the average *FC*_i_ is 96.47 ml accounting for 36.8% of the average *FCs* on intersections; the average *FC*_u_ is 28.05 ml accounting for 10.7% of the average *FCs* on intersections and which is the smallest portion. In other words, 78.4% of the *FCs* on intersections was used at the stages of acceleration and idling.

Meanwhile, different intersection *FC*s are computed and listed (S5 Table).

According to the data showed (S4 Table), *FC* for the No. 6 intersection is 65.13 ml and occupies 24.82% of the *FC* for all intersections and 12.50% of the *FC* for the test route; *FC* for the No. 3 intersection is the second place; and *FC* for the No. 2 intersection is the last one among the all signal control intersections. However, the detailed causes for the high *FC* for the specific intersection will be discussed following.

## Discussions

### Vehicular operating status analysis at an intersection

#### Theoretical analysis

Commonly, the vehicular operating status (VOS) at an intersection can be reduced as three typical types (S4 Fig).

For type (a), the vehicle gradually decelerates to stop behind the stop line or queue behind other vehicles when it faces the red light or the green light but cannot deal with all queuing vehicles of the approach. In general, the vehicle will have a process of deceleration-stop-acceleration to pass through the intersection.

For type (b), the vehicle passes the intersection with a uniform velocity approximately or with a process of slight deceleration-acceleration but without stop. This is one of the objectives of the signal control for all vehicles through the intersection and is favorable for saving *FC*.

For type (c), the vehicle is unable to pass the intersection during the period of one signal cycle. So, the vehicle will suffer from several cycles of deceleration-stop-acceleration and the VOS is the most unstable as well. As a matter of course, it’s the worst control strategy and the most unfavorable for saving *FC*.

#### Experimental data analysis

According to data deduction, the space-velocity diagram for the test vehicle at a specific intersection can be drawn and showed following. Here, a local adjustment is done, but all roads lead to Rome; the distance is used instead of the time for horizontal axis because of more clearness for showing the vehicular operating status on different locations of the intersection.

It shows the space-velocity diagram of the test vehicle at the No. 2 intersection (S5 Fig). The test vehicle passes this intersection as the type (b) of the VOS for fourteen times, three times as the type (a) and once as the type (c). This is a relatively ideal one of the VOS at the intersection and beneficial to saving *FC*.

It shows the space-velocity diagram of the test vehicle at the No. 5 intersection (S6 Fig). It shows that the test vehicle passes the intersection as the type (a) of the VOS mainly, and it’s not so bad for saving *FC*.

It shows the space-velocity diagram of the test vehicle at the No. 6 intersection (S7 Fig). It indicates that the test vehicle passes the intersections as the type (c) of the VOS mainly, and it goes against saving *FC*.

### Causes and improvement strategies of the high *FC* for a specific intersection

#### Causes analysis

Causes of the high *FC* for a specific intersection are basically longer idling time, acceleration time and higher stop frequency. These three factors determine the *FC* for the test vehicle on a specific intersection chiefly, and it can be significantly showed (S6 Table).

As for the No. 6 intersection, there are 2 left-turn pocket lanes, 4 through lanes and 1 right-turn lane at the test route approach and 5 lanes at the exit; there are 2 left-turn pocket lanes, 2 through lanes and 1 right-turn lane at the approach and 3 lanes at test route exit. This intersection is very big actually, and the traffic demand is huge.

$$FC_{c}=10.58S_{f}+0.1t_{i}+21.66(R^{2}=0.99)$$ (9)

Where, *FC*_*c*_ is the *FC* on an intersection; and *S*_*f*_ is the stop frequency.

The index of the stop frequency is of great importance for the computing *FC* on a specific intersection; and the idling time is also significant because 36.7% of the *FC* is wasted.

Stop frequency equals acceleration times. 2 diagrams (S8 and S9 Figs) will show the relationships between *FC* and velocity or acceleration.

From 2 diagrams (S8 and S9 Figs) and the table (S1 Table), it significantly shows that *FC* is so close with the positive acceleration. So, higher stop frequency means much more acceleration times and more *FC*.

#### Improvement strategies

Here, some relative improvement strategies will be present for reducing vehicle *FC* on the area of the intersection. The detailed methods and proof schemes will be discussed in the next article.

First of all, traditional gasoline-powered vehicles should be replaced by the smaller and more efficient vehicles, i.e., hybrid, electric, biofuel, natural gas and etc. Meanwhile, STT for the engine can be promoted because it can reduce *FC* up to 15% during the intersection area. Otherwise, reducing vehicle arrivals in approaches is the fundamental way from changing private cars to bus transit. These are of great importance for sustainable development of arterial road traffic and saving *FC*.

Secondly, the intersections need to be broadened and well channelized for the approaches specially. Of course, it should be combined with the signal control design, especially with the phase design. The number of approach lanes is suggested to equal twice of the number of the road segment lanes; the classification of the lane function should be combined with the vehicle arrival rates; main traffic flow should be identified and should not change lanes frequently; the intersection area should be compressed as small as possible.

And then, optimized signal control strategies should be implemented for the arterial road intersections, for instance, multi-hour signal control schemes for the peak hours and non-peak hours, adaptive signal control method, and signal linear control technique; what’s more, *FC* should be one of the optimization indexes in the control system, and the detailed treatment adopted is that the volume to capacity ratio (V/C) should be lower than 0.8 for the approaches of the intersection. Meanwhile, a pre-signal could be used to the waiting area for through or left-turn traffic. Originally, waiting area setting is beneficial for increasing the intersection capacity, but increases the stop frequency and *FC* at the same time. However, a pre-signal is ahead of the next phase for through or left-turn traffic that the vehicles are allowed to enter the waiting area. Therefore, the vehicles enter the waiting area without extra stops and can save *FC*. Otherwise, the length of the waiting area should exceed 3 vehicles long distance.

Finally, a dynamic velocity control method can be used for the intersection. Here, 2 variable message signs (VMSs) and 3 groups of sensors will be installed along every approach of the intersection. So, the VOS is expected to be adjusted from type (a) to type (b) or from type (c) to type (b). The optimal objective of *FC* on the intersection and the decision variable of the VMS locations were brought forward; then, the optimal model of the dynamic velocity control was proposed after considering the distance, operating speed, vehicle arrival and other constraints; finally, a genetic algorithm was used to solve the model.

### Future research prospects

Finally we gave an outlook to the future research. To begin with, we will conduct much more experimental tests with kinds of vehicles including new energy vehicles, buses and trucks. Moreover, *FC* study will be made under the background of connected vehicles and intelligent transportation systems (ITS) for the signalized intersections. Finally, *FC* for the whole intersection for the entire vehicles under different signal control strategies and different traffic states.

## Conclusions

The preliminary findings are as follows:

1. A test scheme of the *FC* was designed and the experiment was conducted on October, 2014 and July, 2015. So, 18 groups of experimental data were acquired including time, location, speed, engine velocity, engine load, injection timing of the test vehicle.
2. *FC* composing and calculating method was put forward and the *FCR*s were calibrated. First, *FC* has four components, which are *FC*_*a*_, *FC*_*d*_, *FC*_*i*_, and *FC*_*u*_; these four parts are calculated by the recorded segment counts, time interval and corresponding *FCR* respectively. Then, the *FCR*s were calibrated by the collected data, and *f*_a_ is the highest and it’s almost twice of *f*_u_ and four times more of *f*_d_ and *f*_i_. Finally, the *FC* of the test vehicle along the test route was calculated and the average value was 521.11 ml.
3. The characteristics of *FC*s were analyzed for the intersections along the test route. For a start, the definitions of the IID and IOT were given, and the average values were 1,262 meters and 625 seconds which accounted for 28.9% and 68.5% of the whole test route separately. Moreover, the percentages of the *FC*s on intersections in *FC*s were from 42.35% to 59.93% and the average percentage was 50.36%, and it means that more than half of the *FC* was used at the area of intersections compared with 28.9% of the IIDs. Eventually, the average *FC*_a_ was 109.05 ml accounting for 41.6% of the average *FC* on intersections; the average *FC*_i_ was 96.47 ml accounting for 36.8% of the average *FCs* on intersections; in other words, 78.4% of the *FCs* on intersections was used at the stages of acceleration and idling and the key influencing intersections were identified.
4. VOS and causes of the high *FC* for a specific intersection were discussed. Above all, VOS consisted of three types and was illustrated by the collected data. The next, the indexes of idling time, positive acceleration and its times were proved to be the most significant factors on *FC* on intersections. Final, four improvement strategies were proposed for saving *FC* on the area of the intersection.

## Supporting Information

S1 Dataset Dataset. (XLS)

S1 Fig Test route at Harbin, China. (TIF)

S2 Fig The operation interface and the cable of the fuel consumption test system. (TIF)

S3 Fig A space-time diagram of the test vehicle along the test route. (TIF)

S4 Fig Vehicular operating status at an intersection. (TIF)

S5 Fig A space-velocity diagram of the test vehicle at the No. 2 intersection. (TIF)

S6 Fig A space-velocity diagram of the test vehicle at the No. 5 intersection. (TIF)

S7 Fig A space-velocity diagram of the test vehicle at the No. 6 intersection. (TIF)

S8 Fig A time-velocity-fuel consumption diagram of the type (a) of the VOS. (TIF)

S9 Fig A velocity-acceleration-fuel consumption diagram of the type (a) of the VOS. (TIF)

S1 Table Technical Parameters of the Test Vehicle. (DOC)

S2 Table Average FCRs of the Test Vehicle for the Test Route. (DOC)

S3 Table Fuel Consumptions of the Test Vehicle for the Test Route. (DOC)

S4 Table Fuel Consumptions on Intersections. (DOC)

S5 Table Fuel Consumption for a Single Intersection. (DOC)

S6 Table Characteristics of the Test Vehicular Operation and FC on the No. 6 Intersection. (DOC)

## Nomenclature

*FC*: Fuel consumption
*FC_a_*: Acceleration *FC*
*FC_d_*: Deceleration *FC*
*FC_i_*: Idling *FC*
*FC_u_*: Uniform velocity travelling *FC*
*FC_c_*: *FC* on an intersection
*f_a_*: Average acceleration *FC* rate
*f_d_*: Average deceleration *FC* rate
*f_i_*: Average idling *FC* rate
*f_u_*: Average uniform velocity travelling *FC* rate
*q_i_*: Fuel injection quantity for the *i* ^th^ time interval
*τ_i_*: Injection pulse width for the *i* ^th^ record
*C*: Number of cylinders
*K*: Parameter of the fuel injection quantity
*t_a_*: Average acceleration time
*t_d_*: Average deceleration time
*t_i_*: Average idling time
*t_u_*: Average uniform velocity travelling time
*t*_0_: Record time interval
*t*: Time variable
*t_e_*: Injection time interval
*n_a_*: Record acceleration segment count
*n_d_*: Record deceleration segment count
*n_i_*: Record idling segment count
*n_u_*: Record uniform velocity travelling segment count
*n_e_*: Engine velocity
*d*: Density of the gasoline
*S_f_*: Stop frequency for the vehicles
