# Supplementary figures and images for: The Influence of Intersections on Fuel Consumption in Urban Arterial Road Traffic: A Single Vehicle Test in Harbin, China
Source: PLoS One. 2015 Sep 14;10(9):e0137477. doi: 10.1371/journal.pone.0137477 (PMC4569072; doi:10.1371/journal.pone.0137477)

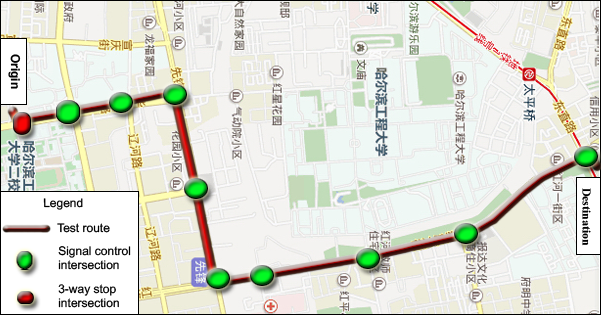

Supplement: S1 Fig — (TIF) [file pone.0137477.s002.tif]

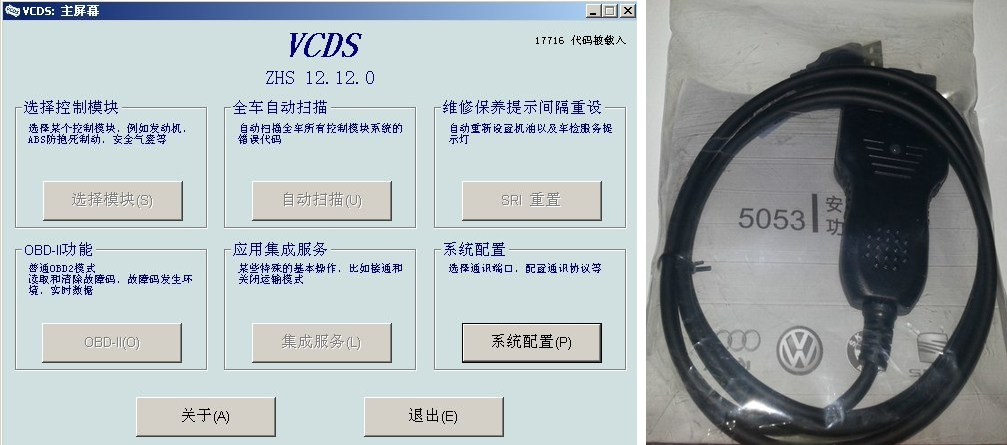

Supplement: S2 Fig — (TIF) [file pone.0137477.s003.tif]

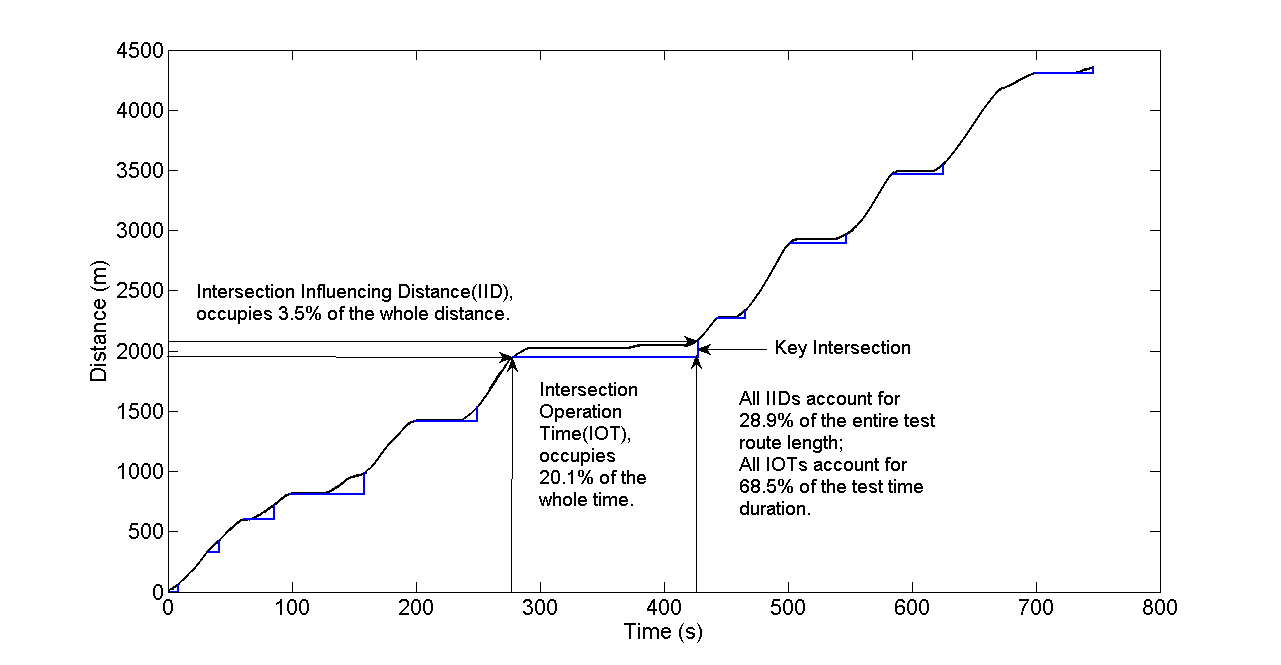

Supplement: S3 Fig — (TIF) [file pone.0137477.s004.tif]

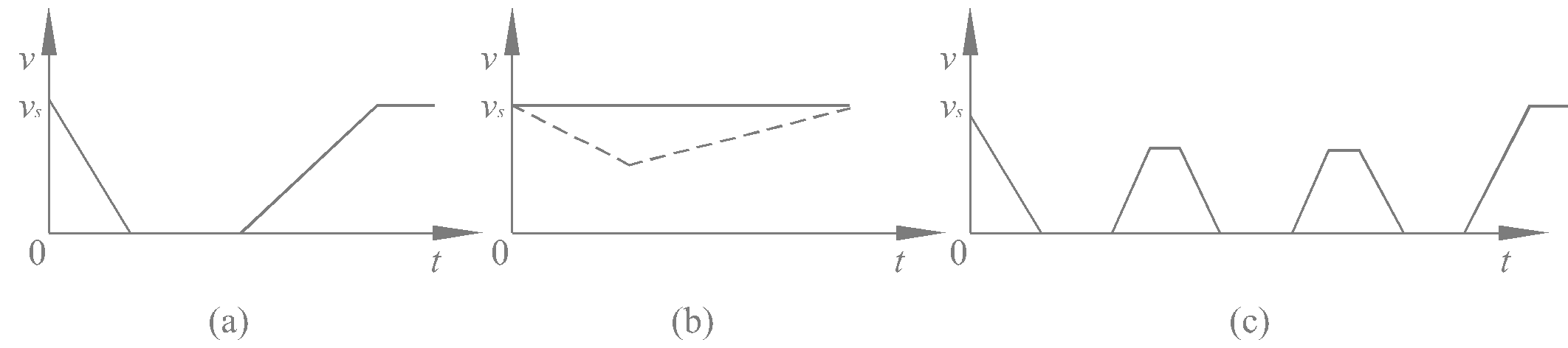

Supplement: S4 Fig — (TIF) [file pone.0137477.s005.tif]

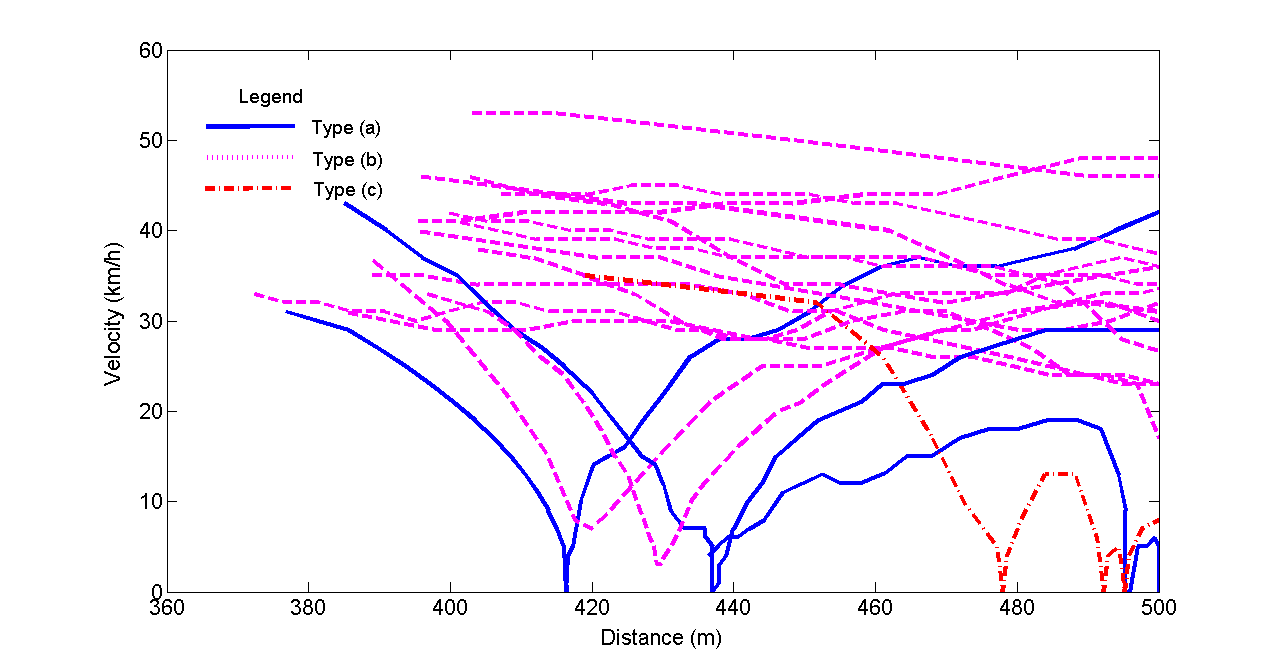

Supplement: S5 Fig — (TIF) [file pone.0137477.s006.tif]

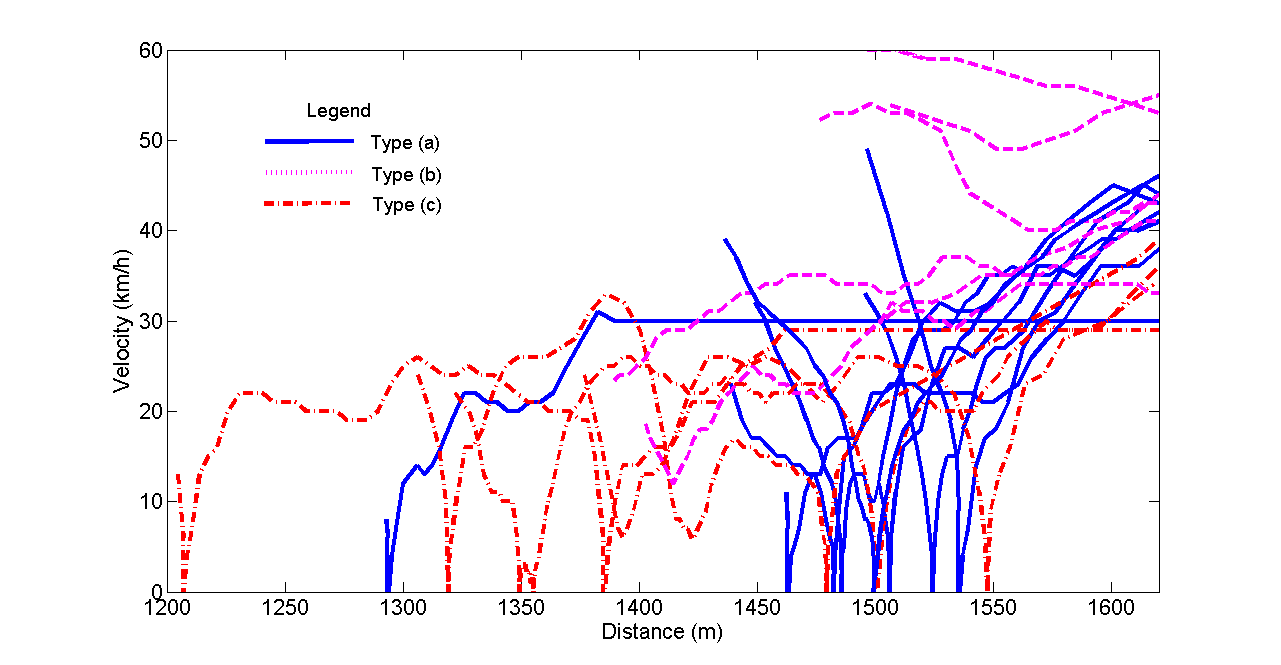

Supplement: S6 Fig — (TIF) [file pone.0137477.s007.tif]

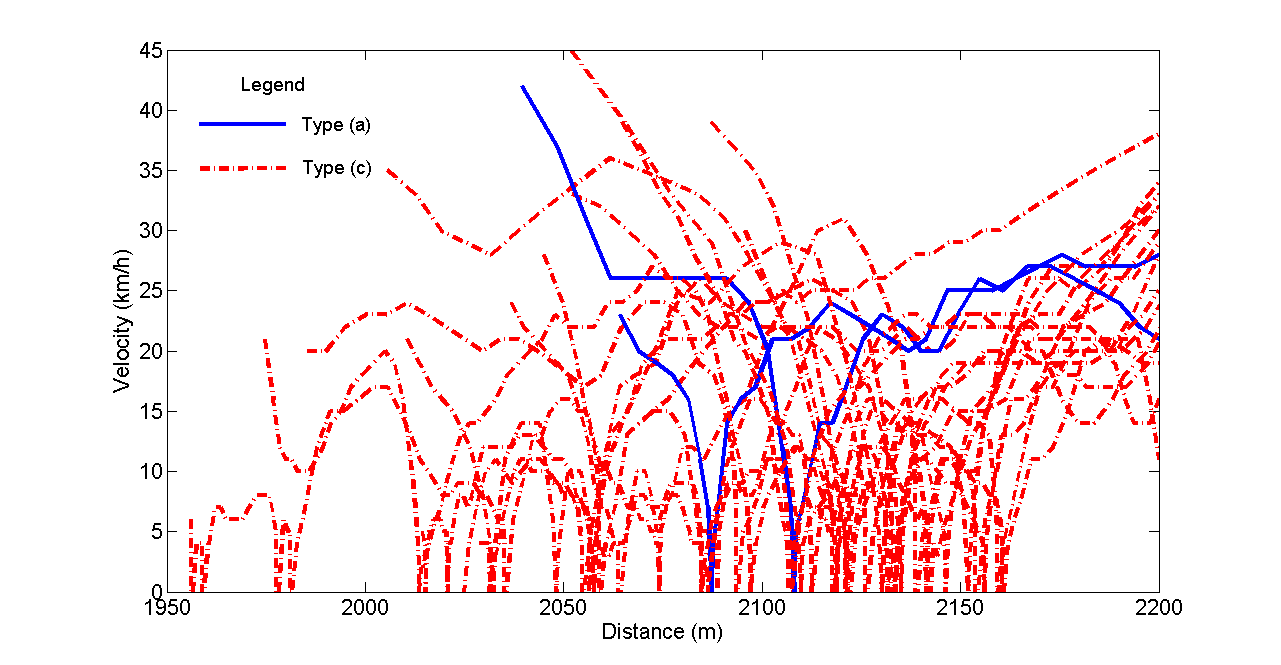

Supplement: S7 Fig — (TIF) [file pone.0137477.s008.tif]

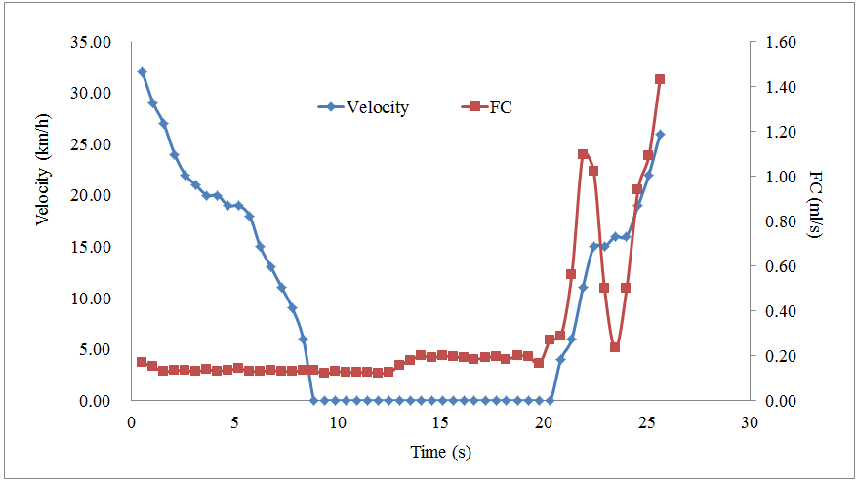

Supplement: S8 Fig — (TIF) [file pone.0137477.s009.tif]

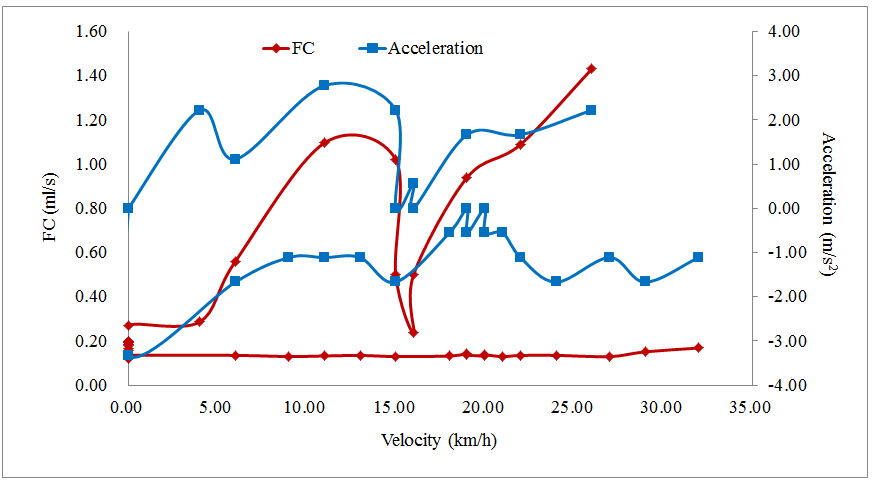

Supplement: S9 Fig — (TIF) [file pone.0137477.s010.tif]
