# Supplementary material for: The Influence of Intersections on Fuel Consumption in Urban Arterial Road Traffic: A Single Vehicle Test in Harbin, China
Source: PLoS One. 2015 Sep 14;10(9):e0137477. doi: 10.1371/journal.pone.0137477 (PMC4569072; doi:10.1371/journal.pone.0137477)
Supplement: S1 Table — (DOC) [file pone.0137477.s011.doc]

**S1 Table. Technical Parameters of the Test Vehicle.**

| **Parameter** | **Property** | **Parameter** | **Property** |
| --- | --- | --- | --- |
| Length | 4428 mm | Length | 4569 mm |
| Width | 1660 mm | Width | 1769 mm |
| Height | 1420 mm | Height | 1462 mm |
| Curb weight | 1050 kg | Curb weight | 1322 kg |
| Real weight | 1200 kg | Real weight | 1472 kg |
| Engine displacement | 1.6 L | Engine displacement | 1.6 L |
| Cylinders | 4 | Cylinders | 4 |
| Compression ratio | 9.30:1 | Compression ratio | 10.5:1 |
| Fuel type | gasoline | Fuel type | gasoline |
| Air intake mode | normally aspirated | Air intake mode | normally aspirated |
| Maximum power rating | 64 kw | Maximum power rating | 77 kw |
| Supply oil style | multi-spot electricity spurts | Supply oil style | multi-spot electricity spurts |
| Transmission | manual | Transmission | automatic |
