# Supplementary material for: The Influence of Intersections on Fuel Consumption in Urban Arterial Road Traffic: A Single Vehicle Test in Harbin, China
Source: PLoS One. 2015 Sep 14;10(9):e0137477. doi: 10.1371/journal.pone.0137477 (PMC4569072; doi:10.1371/journal.pone.0137477)
Supplement: S2 Table — (DOC) [file pone.0137477.s012.doc]

**S2 Table.** **Average *FCR*s of the Test Vehicle for the Test Route.**

| **No.** | ***f_a_* (ml/s)** | ***f_d_* (ml/s)** | ***f_i_* (ml/s)** | ***f_u_* (ml/s)** |
| --- | --- | --- | --- | --- |
| 1 | 1.43 | 0.26 | 0.26 | 0.58 |
| 2 | 1.42 | 0.26 | 0.26 | 0.58 |
| 3 | 1.13 | 0.36 | 0.33 | 0.59 |
| 4 | 1.06 | 0.30 | 0.26 | 0.49 |
| 5 | 1.30 | 0.33 | 0.26 | 0.66 |
| 6 | 1.51 | 0.28 | 0.26 | 0.84 |
| 7 | 1.25 | 0.39 | 0.38 | 0.70 |
| 8 | 1.40 | 0.28 | 0.25 | 0.61 |
| 9 | 1.46 | 0.29 | 0.26 | 0.64 |
| 10 | 1.30 | 0.38 | 0.26 | 0.69 |
| 11 | 1.59 | 0.21 | 0.25 | 0.59 |
| 12 | 1.33 | 0.32 | 0.26 | 0.65 |
| 13 | 1.28 | 0.43 | 0.29 | 0.68 |
| 14 | 1.41 | 0.35 | 0.37 | 0.82 |
| 15 | 1.49 | 0.31 | 0.37 | 0.64 |
| 16 | 1.48 | 0.30 | 0.33 | 0.73 |
| 17 | 1.49 | 0.30 | 0.34 | 0.69 |
| 18 | 1.54 | 0.35 | 0.36 | 0.76 |
| Ave. | 1.38 | 0.32 | 0.30 | 0.66 |
