# Supplementary material for: The Influence of Intersections on Fuel Consumption in Urban Arterial Road Traffic: A Single Vehicle Test in Harbin, China
Source: PLoS One. 2015 Sep 14;10(9):e0137477. doi: 10.1371/journal.pone.0137477 (PMC4569072; doi:10.1371/journal.pone.0137477)
Supplement: S3 Table — (DOC) [file pone.0137477.s013.doc]

**S3 Table. Fuel Consumptions of the Test Vehicle for the Test Route.**

| No. | Test *FC* (ml) | *FC* per hundred kilometers(l/100km) |
| --- | --- | --- |
| 1 | 470.16 | 10.8 |
| 2 | 438.77 | 10.0 |
| 3 | 653.60 | 15.0 |
| 4 | 610.41 | 14.0 |
| 5 | 547.06 | 12.5 |
| 6 | 449.66 | 10.3 |
| 7 | 524.38 | 12.0 |
| 8 | 473.94 | 10.8 |
| 9 | 469.14 | 10.7 |
| 10 | 478.82 | 11.0 |
| 11 | 440.65 | 10.1 |
| 12 | 534.11 | 12.2 |
| 13 | 617.31 | 14.1 |
| 14 | 577.15 | 13.2 |
| 15 | 586.50 | 13.4 |
| 16 | 492.68 | 11.3 |
| 17 | 491.31 | 11.2 |
| 18 | 524.37 | 12.0 |
| Ave. | 521.11 | 11.9 |
