# Supplementary material for: The Influence of Intersections on Fuel Consumption in Urban Arterial Road Traffic: A Single Vehicle Test in Harbin, China
Source: PLoS One. 2015 Sep 14;10(9):e0137477. doi: 10.1371/journal.pone.0137477 (PMC4569072; doi:10.1371/journal.pone.0137477)
Supplement: S4 Table — (DOC) [file pone.0137477.s014.doc]

**S4 Table. Fuel Consumptions on Intersections.**

| No. | Intersections  *FC*_a_ (ml) | Intersections  *FC*_d_ (ml) | Intersections  *FC*_i_ (ml) | Intersections  *FC*_u_ (ml) | Intersections  *FC* (ml) | *FC* (ml) |
| --- | --- | --- | --- | --- | --- | --- |
| 1 | 96.13 | 23.85 | 77.16 | 33.77 | 230.91 | 470.16 |
| 2 | 97.60 | 19.23 | 56.50 | 28.79 | 202.12 | 438.77 |
| 3 | 114.75 | 40.36 | 175.12 | 40.02 | 370.25 | 653.60 |
| 4 | 117.12 | 40.54 | 173.47 | 34.67 | 365.80 | 610.41 |
| 5 | 121.69 | 31.21 | 116.17 | 29.96 | 299.03 | 547.06 |
| 6 | 92.49 | 20.54 | 64.99 | 12.39 | 190.41 | 449.66 |
| 7 | 95.77 | 37.51 | 117.85 | 28.63 | 279.76 | 524.38 |
| 8 | 104.40 | 22.61 | 73.60 | 24.56 | 225.17 | 473.94 |
| 9 | 103.36 | 21.26 | 67.19 | 16.78 | 208.59 | 469.14 |
| 10 | 112.36 | 28.92 | 75.19 | 21.11 | 237.58 | 478.82 |
| 11 | 85.95 | 17.96 | 76.27 | 12.42 | 192.60 | 440.65 |
| 12 | 120.76 | 37.12 | 105.08 | 24.16 | 287.12 | 534.11 |
| 13 | 153.44 | 43.62 | 133.71 | 22.85 | 353.62 | 617.31 |
| 14 | 140.71 | 33.33 | 87.15 | 40.41 | 301.60 | 577.15 |
| 15 | 111.95 | 34.18 | 122.43 | 37.19 | 305.74 | 586.50 |
| 16 | 104.68 | 21.66 | 58.61 | 39.43 | 224.38 | 492.68 |
| 17 | 77.07 | 20.26 | 78.28 | 32.67 | 208.28 | 491.31 |
| 18 | 112.68 | 25.39 | 77.66 | 25.05 | 240.79 | 524.37 |
| Ave. | 109.05 | 28.86 | 96.47 | 28.05 | 262.43 | 521.11 |
