# Supplementary material for: The Influence of Intersections on Fuel Consumption in Urban Arterial Road Traffic: A Single Vehicle Test in Harbin, China
Source: PLoS One. 2015 Sep 14;10(9):e0137477. doi: 10.1371/journal.pone.0137477 (PMC4569072; doi:10.1371/journal.pone.0137477)
Supplement: S5 Table — (DOC) [file pone.0137477.s015.doc]

**S5 Table.** **Fuel Consumption for a Single Intersection.**

| Intersection No. | Intersection *FC* (ml) | Percentage (%) |
| --- | --- | --- |
| 1 | 8.92 | 3.40 |
| 2 | 10.28 | 3.92 |
| 3 | 37.80 | 14.40 |
| 4 | 20.29 | 7.73 |
| 5 | 36.11 | 13.76 |
| 6 | 65.13 | 24.82 |
| 7 | 17.95 | 6.84 |
| 8 | 32.32 | 12.31 |
| 9 | 16.86 | 6.42 |
| 10 | 16.77 | 6.39 |
| Total | 262.43 | 100.00 |
