# Supplementary material for: The Influence of Intersections on Fuel Consumption in Urban Arterial Road Traffic: A Single Vehicle Test in Harbin, China
Source: PLoS One. 2015 Sep 14;10(9):e0137477. doi: 10.1371/journal.pone.0137477 (PMC4569072; doi:10.1371/journal.pone.0137477)
Supplement: S6 Table — (DOC) [file pone.0137477.s016.doc]

**S6 Table. Characteristics of the Test Vehicular Operation and *FC* on the No. 6 Intersection.**

| Test No. | Stop  frequency | Idling  time(s) | *FC*_i_ (ml) | Acceleration  time (s) | *FC*_a_ (ml) | *FC* (ml) |
| --- | --- | --- | --- | --- | --- | --- |
| 1 | 4 | 143 | 38 | 17 | 15.20 | 72.50 |
| 2 | 2 | 81 | 20 | 11 | 15.00 | 41.00 |
| 3 | 4 | 150 | 46 | 24 | 24.40 | 85.70 |
| 4 | 2 | 102 | 25 | 14 | 17.80 | 50.10 |
| 5 | 8 | 341 | 87 | 31 | 33.80 | 138.50 |
| 6 | 3 | 145 | 38 | 18 | 22.10 | 66.10 |
| 7 | 2 | 101 | 37 | 18 | 15.40 | 66.60 |
| 8 | 1 | 101 | 26 | 11 | 13.70 | 46.10 |
| 9 | 2 | 116 | 30 | 11 | 11.80 | 49.60 |
| 10 | 2 | 100 | 25 | 12 | 15.30 | 45.90 |
| 11 | 1 | 79 | 20 | 8 | 10.20 | 38.40 |
| 12 | 5 | 222 | 55 | 25 | 32.00 | 97.60 |
| 13 | 2 | 109 | 28 | 20 | 18.10 | 50.10 |
| 14 | 3 | 129 | 49 | 29 | 31.00 | 100.05 |
| 15 | 2 | 90 | 35 | 14 | 14.51 | 60.47 |
| 16 | 2 | 22 | 7 | 16 | 17.77 | 40.95 |
| 17 | 3 | 88 | 32 | 15 | 18.45 | 60.63 |
| 18 | 2 | 68 | 27 | 19 | 23.89 | 62.15 |
| Ave. | 3 | 121 | 35 | 17 | 19.47 | 65.14 |
